# Draft Genome Sequence of *Methylobacterium radiotolerans, *a DDE-Degrading and Plant Growth-Promoting Strain Isolated from *Cucurbita pepo*

**DOI:** 10.1128/genomeA.00488-15

**Published:** 2015-05-14

**Authors:** Nele Eevers, Jonathan D. Van Hamme, Eric M. Bottos, Nele Weyens, Jaco Vangronsveld

**Affiliations:** a Centre for Environmental Sciences, Hasselt University, Diepenbeek, Belgium; b Department of Biological Sciences, Thompson Rivers University, Kamloops, British Columbia, Canada

## Abstract

We announce the draft genome of *Methylobacterium radiotolerans*, a Gram-negative bacterium isolated from *Cucurbita pepo* roots. This strain shows 2,2-bis(*p*-chlorophenyl)-1,1-dichloroethylene (DDE)-degrading potential and plant growth-promoting capacities. Analyses of its 6.8-Mb genome will improve our understanding of DDE-degradation pathways and aid in the deployment of phytoremediation technologies to remediate DDE-contaminated soils.

## GENOME ANNOUNCEMENT

DDT [2,2-bis(*p*-chlorophenyl)-1,1,1-trichloroethane] (1) is a recalcitrant bioaccumulative pesticide introduced in 1943 that has been used for agriculture, for gardening, and to control the spread of diseases such as malaria (2). The primary degradation product in soil, 2,2-bis(*p*-chlorophenyl)-1,1-dichloroethylene (DDE), is classified as a persistent organic pollutant (POP) along with DDT (3). These materials are hormone disrupting and can be acutely and chronically toxic to wildlife (4). Endophyte-enhanced phytoremediation using the DDE-accumulating zucchini plant, *Cucurbita pepo* (5), is currently being explored for soil remediation.

Here, we describe a whole-genome shotgun sequence for *Methylobacterium radiotolerans*, a DDE-degrading strain isolated from the roots of *C. pepo* plants that were grown with 100 mg·liter^−1^ of DDE. The strain was identified by partial 16S rRNA gene sequencing, and the closest related 16S rRNA sequence (99%) was from strain JCM2831 (GenBank accession no. CP001001.1).

An IonTorrent PGM was used to generate a whole-genome shotgun sequence for *M. radiotolerans* using methods described by Eevers et al. (6).

A total of 379 Mb of data were generated in Torrent Suite version 4.2.1 and assembled into 240 contigs >1,000 bp using SPAdes version 3.1.0 (7, 8) (uniform coverage mode; *k*-mers 21, 33, 55, 77, 99), giving a consensus length of 6,810,393 bp at a 29× coverage (largest contig, 210,399 bp; *N*_50_, 57,566 bp). Contigs were ordered with the genome of *Methylobacterium radiotolerans* JCM2831 as a reference in Mauve (9). Annotation was completed using the PGAP (NCBI) pipeline (10). The genome of *M. radiotolerans* consists of a single circular chromosome (71.19% GC content), including 6,373 coding genes that were arranged into 338 pathways with Pathway Tools (11, 12), 1,474 pseudogenes, 13 rRNAs (5S, 16S, 23S), 50 tRNAs, and 1 noncoding RNA (ncRNA).

In laboratory experiments, *Methylobacterium radiotolerans* exhibited increased growth yields when DDE was added to the medium. Analyses of the draft genome revealed the presence of halogenases, dioxygenases, and hydrolases that have been previously reported to be implicated in DDE degradation (13–16). *Methylobacterium radiotolerans* also possesses genes associated with the degradation of toluene, naphthalene, acrylonitrile, phenylacetate, and nitriloacetate, as well as genes for arsenate detoxification. Genes that code for plant growth-promoting capacities, such as 1-aminocyclopropane-1-carboxylate deaminase activity, siderophore production, auxin biosynthesis, and phosphorous solubilization, are present, and these activities were confirmed *in vitro*. Furthermore, the genome harbors genes for nitrogen fixation, glutathione biosynthesis, and the degradation of superoxide radicals. Given its biodegradative and plant growth-promoting abilities, *Methylobacterium radiotolerans* is being studied as an inoculant for the phytoremediation of DDE-contaminated soils.

### Nucleotide sequence accession numbers.

This whole-genome shotgun project has been deposited at DDBJ/EMBL/GenBank under the accession number JXTO00000000. The version described in this paper is version JTXO01000000.
